# Cross-sectional study of citations from Brazilian Vascular Journal and Brazilian authors between the years 2016-2024

**DOI:** 10.1590/1677-5449.202500162

**Published:** 2025-12-15

**Authors:** Lucas Peclat, Lucas Costa Barbosa, Rafael Peclat, Julio Cesar Peclat de Oliveira, Marina Araujo Zulchner, Bianca Gutfilen, Rossano Kepler Alvim Fiorelli, Marcos Arêas Marques

**Affiliations:** 1 Universidade Federal do Estado do Rio de Janeiro – UNIRIO, Rio de Janeiro, RJ, Brasil.; 2 Universidade Federal do Rio de Janeiro – UFRJ, Rio de Janeiro, RJ, Brasil.; 3 Faculdade Técnico Educacional Souza Marques – FTESM, Rio de Janeiro, RJ, Brasil.; 4 Clínica Peclat, Rio de Janeiro, RJ, Brasil.

**Keywords:** publications, open access publishing, scholarly communication, citation database, journal index, periodical publications

## Abstract

**Background:**

Although the overall volume of Brazilian scientific research has increased over the past decades, for years there has been a noticeable lack of prestige attributed to national publications in favor of foreign journals. This dynamic has limited their influence and the development of Brazilian lines of research.

**Objectives:**

To analyze the references used by the Brazilian Vascular Journal (BVJ), in relation to its own journal of origin, in order to identify the representativeness and relevance of national journals.

**Methods:**

A cross-sectional observational study was carried out, examining 13,633 bibliographic references cited in 582 articles from the BVJ, published between 2016 and 2024 and indexed in PubMed. The references were extracted and organized in a Microsoft Excel^®^ spreadsheet, in which the origin of each citation was verified, with an emphasis on identifying which were Brazilian and/or from the BVJ, in addition to searching for self-citations by the first author.

**Results:**

A total of 1,300 (9.5%) citations of articles from Brazilian journals were identified in the BVJ. The BVJ was the most frequently cited national journal, with 613 references (4.5%). It was observed that 189 (32%) of the articles analyzed did not include any national reference. The percentage of self-citations was 8%, showing a downward trend over time.

**Conclusions:**

The low number of citations is consistent with what has been previously described in the national literature, which has warned of the same issue for 30 years. The decreasing percentage of self-citations may suggest greater diversity of sources used over the years. These findings highlight a culture of disregard for national journals, which hinders original research and compromises the development of research lines focused on the Brazilian population.

## INTRODUCTION

"If I have seen further, it is by standing on the shoulders of giants." This famous excerpt from a letter by Sir Isaac Newton, while popular and apparently simple at first glance, cloaks in its few brief words an immense baggage that is illustrative of the whole of science. Dialectic (thesis, antithesis, and synthesis) has always been part of the process of construction of knowledge and development of research streams by conflicting ideas is as essential as the scientific method itself.^1^

The number of Brazilian publications has increased significantly over recent decades, from 9,600 articles published annually, in 1996, to more than 80,000 over recent years.^2^ This growth can be attributed to greater ease of access to scientific research and to incremental incentives for engaging in Brazilian science, which has the capability to deliver innovative and specific solutions to the population’s needs. However, although the volume of scientific work is of fundamental importance for a country’s production of high-quality knowledge, it does not necessarily lead to creation of new research streams targeting local problems. To achieve this, it is necessary to publicize scientific discoveries via appropriate channels, so they will precisely hit their target audiences and spawn more debates and ideas.

The Brazilian Vascular Journal (BVJ) is an open-access Brazilian publication indexed on many different databases such as, for example, SciELO, PubMed, Web of Science, Scopus, Literatura Latino-Americana e do Caribe em Ciências de Saúde (LILACS), EBSCO, Embase, and the Directory of Open Access Journals. It has been published by the Brazilian Society of Angiology and Vascular Surgery (SBACV - Sociedade Brasileira de Angiologia e de Cirurgia Vascular) since 2002, in Portuguese and English.

The mission of the BVJ is to select and publish high-quality scientific articles on original primary or secondary studies, new surgical and diagnostic techniques, and clinical observations in the areas of vascular surgery, angiology, and endovascular surgery, in addition to reviews, case reports, brief communications, study protocols, therapeutic challenges, and letters to the editors.

The objective of this study is to analyze the references cited in BVJ articles in terms of citation of Brazilian articles, citation of articles published in the BVJ itself, and author self-citations.

## METHODS

The objective of this cross-sectional observational study was to analyze the references cited in articles published in the BVJ from 2016 to 2024, considering only articles available on the PubMed database up to February of 2024. Self-citations of Brazilian authors in international journals were not counted, since the scope of the study is limited to citations of Brazilian journals and self-citations in the BVJ itself. The study was designed to comply with the Strengthening the Reporting of Observational Studies in Epidemiology (STROBE) guidelines, guaranteeing a transparent and rigorous description of the methodological procedures adopted.

### Inclusion criteria and sampling

The study sampled all BVJ articles indexed on PubMed up to February 2024, with no additional exclusion criteria. It should be noted that, due to the PubMed indexing policy, not all of the articles published in the BVJ since its first edition are available via the database. This limitation constitutes a potential source of sampling bias, since the sample available for analysis may not be entirely representative of the journal’s historical production.

### Search strategy and data collection

Data were collected using a specific search strategy via the PubMed platform, using the search string “’Brazilian Vascular Journal ’[Journal]”. All of the articles returned by this search were selected for the study. Additionally, a supplementary search was run to identify journals hosted in Brazil and indexed on PubMed, using the search string “Brazil [Country]” in the National Library of Medicine (NLM) Catalog. This step was taken in order to identify the origin of each reference cited and thus calculate the proportion of Brazilian journals in the sample.

### Extraction and organization of data 

Data were extracted from the articles and their reference lists using the Biopython library, via the Python framework, which was used to access and download the information available via PubMed. The data downloaded were organized in a Microsoft Excel® spreadsheet and categorized using the following variables: PubMed identifier (PMID); title; journal; publication date; authors; affiliations; number of citations of Brazilian journals; and number of citations of the BVJ.

### References

Each spreadsheet column was carefully analyzed. For example, the “PubMed identifier (PMID)” enables article tracking, while the variables “title”, “journal” and “publication date” provide essential contextual information. The variables “number of citations of Brazilian journals” and “number of citations of the BVJ” were calculated to quantify the proportion of citations that were of Brazilian journals, and the variable “references” was used to conduct a detailed analysis of the set of citations referenced.

### Data verification and validation

In order to ensure the quality and integrity of the data extracted, the automated procedure was supplemented by manual verifications conducted by at least two authors. This process involved crosschecking the data in the spreadsheet against information available on PubMed and in the NLM Catalog, ensuring that the categories and values extracted were consistent and correct.

### Definition and operationalization of variables

Number of citations of Brazilian journals: the total number of citations of articles published in Brazilian journals in the references of the articles analyzed. Number of citations of the BVJ: the total number of citations of articles published in the BVJ itself.

### Self-citations

The analysis of self-citations focused on identifying cases in which lead authors of articles in the BVJ cited articles published in Brazilian journals that they had authored themselves, enabling assessment of the incidence of this phenomenon in the sample.

### Data presentation

Data were presented descriptively, using graphs and tables to facilitate visualization and interpretation of the findings. No inferential statistical analyses were conducted since the primary objective was a descriptive synthesis of the data extracted.

### Limitations and considerations

One important limitation of this study is the fact that PubMed does not index all of the articles published in the BVJ since its first edition. As such, the sample may not reflect the journal’s entire production, which in turn could influence interpretation of the results. Moreover, despite having undergone manual checking, the automated data extraction process is vulnerable to any indexing errors or inconsistencies.

### Ethics

This study is exempt from submission for assessment by the Research Ethics Committee/National Research Ethics Commission (Comissão Nacional de Ética em Pesquisa - CEP/CONEP). According to the CEP/CONEP system, studies that are entirely based on bibliographic data extracted from public databases do not require approval in advance.

## RESULTS

A total of 582 articles published from 2016 to 2024 were extracted, with a total of 13,633 references cited. Observing the distribution of articles, there was no clear linear trend to increases or reductions in the number of articles published per year: the number increased up to 2021 (reaching 110 articles), then reduced over the three years 2022-2024, with predominantly erratic behavior.

There were 1,300 (9.5%) references to articles in Brazilian journals in the total sample, 613 (4.5%) of which were citations of BVJ articles. These citations of Brazilian journals were highly concentrated in 79 of the 372 Brazilian periodicals indexed on PubMed.

The number of citations of Brazilian journals per year remained relatively stable, with a mean of 2.2 per article published in the BVJ. The number of citations of the BVJ also exhibited stable behavior, with a mean of 1.1 per article published in the BVJ (Table 1).

**Table 1:** Trends in citations of Brazilian journals, self-citations, and impact per article in the BVJ (2016-2024).

Year	Brazilian citations	BVJ citations	BVJ self-citations	Self-citations (%)	Number of articles	Brazilian citations per article	BVJ citations per article
2016	108	54	11	20	45	2.4	1.2
2017	109	49	10	20	59	1.8	0.8
2018	120	50	7	14	61	2.0	0.8
2019	177	87	12	14	67	2.6	1.3
2020	181	88	15	17	75	2.4	1.2
2021	225	103	9	9	110	2.0	0.9
2022	144	74	6	8	58	2.5	1.3
2023	148	66	6	9	66	2.2	1.0
2024	88	42	4	10	41	2.1	1.0

BVJ = Brazilian Vascular Journal.

**Figure 1:**
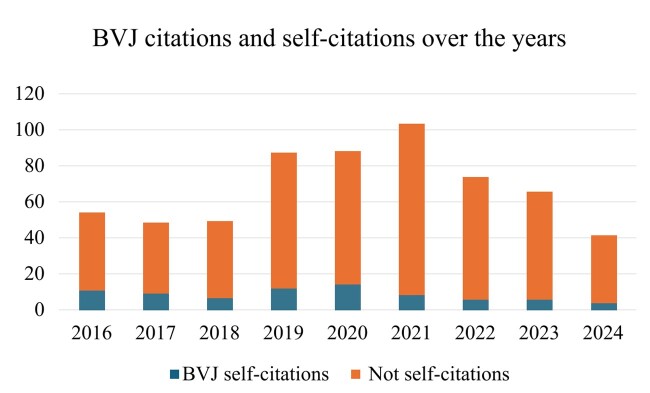
Numbers of citations and self-citations over the years. BVJ = Brazilian Vascular Journal.

Additionally, there were 80 (13%) self-citations by lead authors among the 613 BJV citations. This rate fell progressively over the years, from 20% in 2016 to 8% in 2022 (Figure 1).

**Figure 2:**
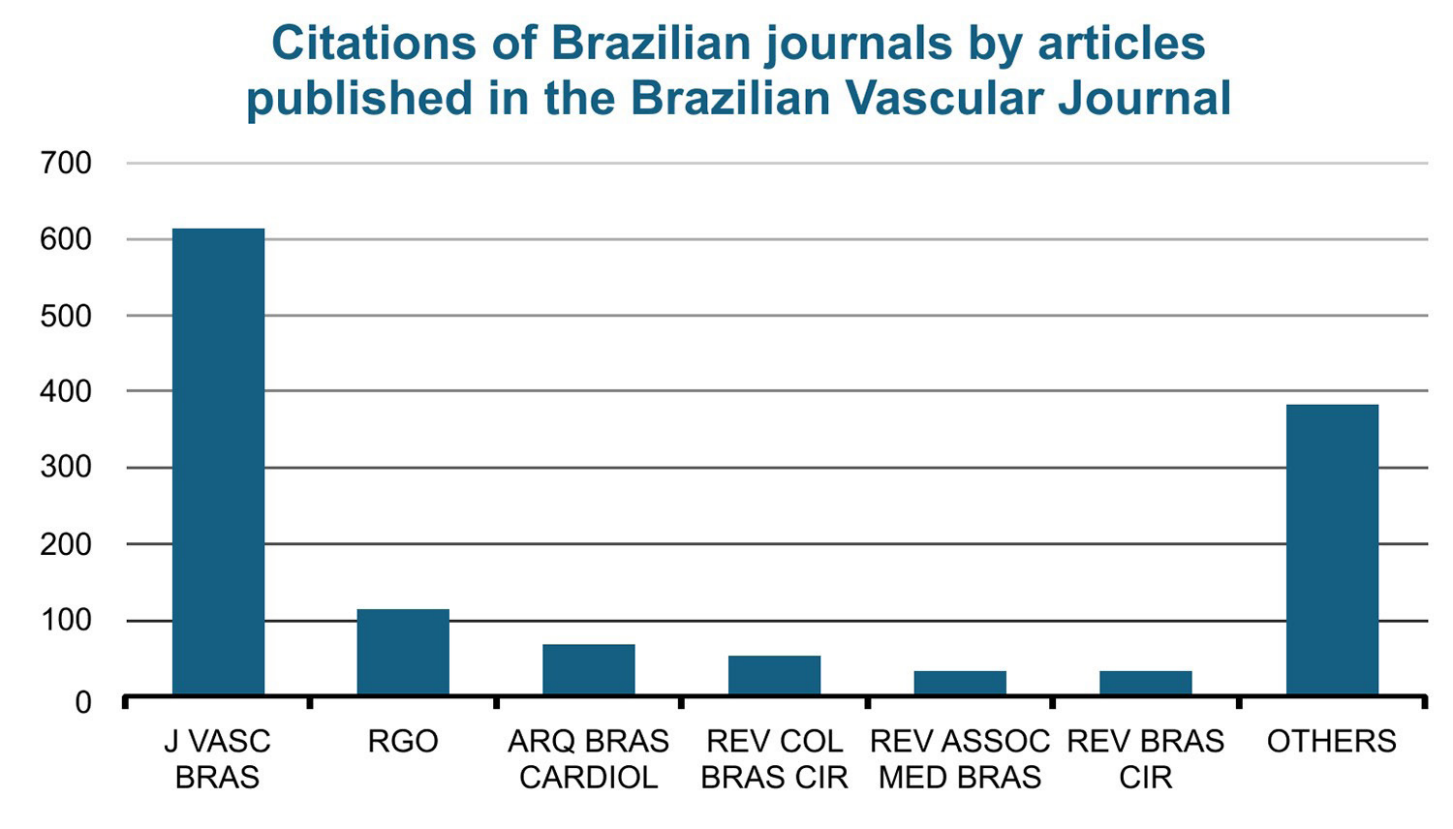
Citations of Brazilian journals by articles published in the Brazilian Vascular Journal from 2016 to 2024. J VASC BRAS = Jornal Vascular Brasileiro (Brazilian Vascular Journal); RGO = Revista Brasileira de Ortopedia; ARQ BRAS CARDIOL = Arquivos Brasileiros de Cardiologia; REV COL BRAS CIR = Revista do Colégio Brasileiro de Cirurgiões; REV ASSOC MED BRAS = Revista da Associação Médica Brasileira; REV BRAS CIR = Revista Brasileira de Cirurgia Plástica.

The Brazilian journals most cited were, in order of prevalence: the BVJ, Revista Brasileira de Ortopedia (RGO), Arquivos Brasileiros de Cardiologia, Revista do Colégio Brasileiro de Cirurgiões, Revista da Associação Médica Brasileira, Revista Brasileira de Cirurgia Plástica, Jornal Brasileiro de Pneumologia, Acta Cirúrgica Brasileira, Radiologia Brasileira, and the Cadernos de Saúde Pública. Journals on surgery and other areas linked to vascular surgery, such as cardiology and respiratory medicine predominated. Notwithstanding, citations of articles from the BVJ itself still accounted for a significant percentage of citations of Brazilian journals, with 47.1% of all such citations (Figure 2).

The majority of the articles analyzed had from 0.01 to 25.00% Brazilian citations in their reference lists. Overall, 189 (32.00%) of the articles analyzed did not cite a single reference published in any of the Brazilian journals indexed on PubMed. Just 11 (1.90%) of the articles analyzed had more than half of their references citing Brazilian journals and none had a reference list entirely comprising Brazilian articles (Table 2).

**Table 2:** Distribution of percentages of citations of Brazilian journal articles in the BVJ.

% of citations of Brazilian journals		
0%	189	32%
0.01-25%	323	55%
25-50%	59	10%
51-75%	10	2%
> 75%	1	0%

BVJ = Brazilian Vascular Journal.

## DISCUSSION

In common with all science, this study builds on foundations laid by colleagues interested in the same subjects. The term scientific voyeurism was coined by Goffi, to describe disdain for Brazilian journals in favor of international publications. The practice does not only do an injustice to Brazilian authors and their efforts to advance Brazilian science, but also hinders the development of journals published domestically in relation to their peers, weakening the image and compromising the development of Brazilian science as a whole.^3^

A Brazilian study conducted by the Brazilian College of Surgeons (Colégio Brasileiro de Cirurgiões) in 2012 reported that 11.65% of 7,000 references in three prominent general surgery journals were to Brazilian sources, which is compatible with the overall percentage of citations observed in the present study. The majority of articles in the BVJ were in the range of 0.01 to 25.00% of Brazilian citations and just 11 articles cited more Brazilian references than international sources in their discussions.^4^

Articles published in orthopedics journals have been drawing attention to this situation for more than 30 years, when it was shown that the RBO had a low percentage of citations of Brazilian references in relation to other journals, even in relation to articles published in the RBO itself.^5^ Publication of that article provoked much debate in the area and letters to the editor were penned sharing the experience of Brazilian authors who had been overlooked in favor of international publications on the same subject.^6^

It is of note that the rate of self-citations reduced progressively, falling to 8% over the period studied. This may be evidence of development of a non-predatory scientific ecosystem, in which authors publish and exchange citations, expanding the reach of Brazilian research.

The decision to publish in a journal involves many different factors. However, metrics such as the impact factor (IF) carry a disproportional weight in the academic world, since they are the principal tool used to assess the quality of scientific journals.^7^ Consequently, publication in journals with a high IF becomes a symbol of “scientific status”, diverting original articles from being published in the most appropriate journals.^8^

International studies indicate that preference for high impact journals is a global phenomenon. Factors such as accessibility, language of publication, and editorial strategies have significant influence on citation patterns. Journals published in English and with open access tend to achieve greater visibility, while regional publications can suffer from reduced recognition and, consequently, lower citation indices.^9^

Several methods to combat this phenomenon have been debated over the years. Faced with a huge contingent of scientists, but an inaccessible language, many Chinese journals began to publish articles simultaneously in two languages: Mandarin Chinese and English. This increases local circulation of information and confers prestige on Chinese organizations, whilst also removing barriers to international researchers who wish to dig deeper into their subjects of study and cite these publications.^10^ It should be pointed out that this practice has always been the case with the BVJ, which publishes all articles in both Portuguese and English.

Campaigns to encourage publication of articles in Brazilian journals have also been conducted by medical societies, such as the SBACV. However, while they have had a social impact, no data are available on the true effectiveness of the strategy in terms of the number of citations and publications of Brazilian studies.

Considering the attractiveness of the international journals, it falls to Brazilian journals to encourage strategies such as, for example, publication of articles on social media or even awarding prizes to authors based on parameters such as number of citations.

The cause of the low rate of citation of Brazilian authors in the BVJ is multifactorial: it reflects a cultural preference for foreign journals (the “mongrel complex”), differences in evidence level between Brazilian studies and foreign prospective and randomized studies, and the need to attain a competitive impact factor (IF > 2.0) to start off a virtuous circle of visibility and citations. This pattern confirms prior studies of the subject,^11^ which have also found evidence of underrepresentation of Brazilian authors in Brazilian surgery journals.

## CONCLUSIONS

This study analyzed 13,633 references from articles published in the BVJ from 2016 to 2024 and found that just 1,300 (9.5%) of these references cited articles in Brazilian journals, 613 (4.5%) of which were published in the BVJ itself. It was also observed that there was a falling trend in the rate of self-citations, which suggests diversification of the sources cited. These findings provide evidence, of a descriptive nature, of the profile of citations in the BVJ and indicate a need for strategies to raise the visibility of Brazilian journals in the scientific literature.

## Data Availability

Os dados que fundamentam os achados deste estudo estão disponíveis no repositório Pubmed ([https://pubmed.ncbi.nlm.nih.gov/).
