# Supplementary material for: Cross-sectional study of citations from Brazilian Vascular Journal and Brazilian authors between the years 2016-2024
Source: J Vasc Bras. 2025 Dec 15;24:e20250016. doi: 10.1590/1677-5449.202500162 (PMC12704809; doi:10.1590/1677-5449.202500162)
Supplement: Translation

# Translation: Estudo transversal das citações do Jornal Vascular Brasileiro e de autores brasileiros entre os anos de 2016-2024

**DOI:** 10.1590/1677-5449.202500161

**Authors:** Lucas Peclat, Lucas Costa Barbosa, Rafael Peclat, Julio Cesar Peclat de Oliveira, Marina Araujo Zulchner, Bianca Gutfilen, Rossano Kepler Alvim Fiorelli, Marcos Arêas Marques

**Affiliations:** 1 Universidade Federal do Estado do Rio de Janeiro – UNIRIO, Rio de Janeiro, RJ, Brasil.; 2 Universidade Federal do Rio de Janeiro – UFRJ, Rio de Janeiro, RJ, Brasil.; 3 Faculdade Técnico Educacional Souza Marques – FTESM, Rio de Janeiro, RJ, Brasil.; 4 Clínica Peclat, Rio de Janeiro, RJ, Brasil.

**Keywords:** publicações, publicação de acesso aberto, comunicação acadêmica, base de dados de citações, índice de periódicos, publicações periódicas

## Abstract

**Contexto:**

Embora o volume total de pesquisa científica brasileira venha aumentando nas últimas décadas, há anos se observa um desprestígio de publicações nacionais em favor das estrangeiras, o que limita sua influência e o desenvolvimento de linhas de pesquisa brasileiras.

**Objetivos:**

Analisar as referências utilizadas pelo Jornal Vascular Brasileiro (JVB), em relação à sua revista de origem, de modo a identificar a representatividade e a relevância dos periódicos nacionais.

**Métodos:**

Foi realizado um estudo observacional transversal que examinou 13.633 referências bibliográficas citadas em 582 artigos do JVB, publicados entre 2016 e 2024 e indexados no PubMed. As referências foram extraídas e organizadas em uma planilha do Microsoft Excel^®^, em que se verificou a origem de cada citação, com ênfase em identificar quais eram brasileiras e/ou do JVB, além de buscar autocitações do primeiro autor.

**Resultados:**

Foram encontradas 1.300 (9,5%) citações de artigos de revistas brasileiras no JVB. O JVB foi o periódico nacional mais citado, com 613 referências (4,5%). Verificou-se que 189 (32,0%) artigos avaliados não utilizaram nenhuma referência nacional. O percentual de autocitações foi de 8,0%, mas apresentou queda ao longo do período.

**Conclusões:**

O baixo número de citações é condizente com o descrito previamente pela literatura nacional, que há 30 anos alerta sobre o problema. O percentual decrescente de autocitações pode sugerir maior diversidade de fontes utilizadas ao longo dos anos. Evidencia-se uma cultura de desprestígio aos periódicos nacionais, afastando pesquisas originais e comprometendo o desenvolvimento de linhas de pesquisa voltadas à população brasileira.

## INTRODUÇÃO

“Se enxerguei mais longe, foi por estar sobre ombros de gigantes”. O famoso trecho da carta de Sir Isaac Newton, embora popular e aparentemente simples à primeira vista, esconde, em suas breves palavras, uma imensa bagagem representativa de toda a ciência. A dialética (tese, antítese e síntese) sempre foi parte do processo de construção de conhecimento, e o desenvolvimento de linhas de pesquisa através do conflito de ideias é tão essencial quanto o próprio método científico em si^1^.

O número de publicações brasileiras aumentou significativamente nas últimas décadas, partindo de 9.600 publicações anuais, em 1996, para mais de 80.000 nos anos recentes^2^. Esse crescimento pode ser atribuído à maior facilidade de acesso às pesquisas científicas e ao incremento do incentivo ao desenvolvimento da ciência brasileira, que pode trazer soluções inovadoras e específicas às demandas da população. Entretanto, embora o volume científico seja de fundamental importância para a produção de conhecimento de qualidade para um país, ele não necessariamente implica na criação de novas linhas de pesquisa direcionadas aos problemas locais. Para tal, é essencial que as descobertas científicas sejam propagadas em canais adequados, a fim de atingir seu público-alvo de forma precisa e levantar mais debates e ideias.

O Jornal Vascular Brasileiro (JVB) é uma publicação brasileira de acesso gratuito, indexada em diversas bases de dados como, por exemplo, SciELO, PubMed, Web of Science, Scopus, Literatura Latino-Americana e do Caribe em Ciências da Saúde (LILACS), EBSCO, Embase e Directory of Open Access Journals. É publicado pela Sociedade Brasileira de Angiologia e de Cirurgia Vascular (SBACV) desde 2002, em língua portuguesa e inglesa.

A missão do JVB é selecionar e disseminar conteúdos de qualidade científica comprovada acerca de estudos originais, primários ou secundários, novas técnicas cirúrgicas e diagnósticas, e observações clínicas nas áreas de cirurgia vascular, angiologia e cirurgia endovascular, assim como revisões, relatos de caso, notas prévias, protocolos de estudo, desafios terapêuticos e cartas aos leitores.

O objetivo deste estudo é avaliar as referências utilizadas pelos artigos do JVB em relação à citação de artigos nacionais, à citação de artigos do próprio JVB e às autocitações.

## MÉTODOS

Este estudo observacional e transversal foi conduzido com o objetivo de analisar as referências utilizadas nos artigos publicados no JVB durante o período de 2016 a 2024, considerando apenas os artigos disponíveis na base de dados PubMed até fevereiro de 2024. As autocitações de autores brasileiros em revistas internacionais não foram computadas, pois o foco deste estudo foi restrito às citações de periódicos nacionais e às autocitações dentro do próprio JVB. O estudo foi desenvolvido em conformidade com as diretrizes Strengthening the Reporting of Observational Studies in Epidemiology (STROBE), garantindo um relato transparente e rigoroso dos procedimentos metodológicos adotados.

### Critérios de inclusão e amostragem

Foram incluídos no estudo todos os artigos do JVB indexados no PubMed até fevereiro de 2024, sem a aplicação de critérios de exclusão adicionais. Ressalta-se que, devido à natureza da indexação do PubMed, nem todos os artigos publicados pelo JVB desde sua primeira edição estão disponíveis na base de dados. Essa limitação constitui um potencial viés de amostragem, pois a amostra analisada pode não representar integralmente a produção histórica do periódico.

### Estratégia de busca e coleta de dados

A coleta dos dados foi realizada por meio de uma estratégia de busca específica na plataforma PubMed, utilizando o termo “’Jornal Vascular Brasileiro’[Journal]”. Todos os artigos resultantes desta busca foram considerados para o estudo. Adicionalmente, foi realizado um levantamento complementar das revistas sediadas no Brasil e indexadas no PubMed, utilizando a estratégia de busca “Brazil [Country]” no National Library of Medicine (NLM) Catalog. Essa etapa permitiu identificar a origem de cada referência e caracterizar a representatividade dos periódicos brasileiros na amostra.

### Extração e organização dos dados

A extração dos dados dos artigos e de suas referências foi automatizada por meio da biblioteca Biopython, do *framework* Python, que possibilitou acessar e baixar as informações disponíveis no PubMed. Os dados extraídos foram organizados em uma planilha do Microsoft Excel® e categorizados nas seguintes variáveis: identificador no PubMed (PMID); título; periódico; data de publicação; autores; afiliações; número de citações de revistas brasileiras; número de citações do JVB.

### Referências

Cada coluna da planilha foi cuidadosamente revisada. Por exemplo, o “identificador no PubMed (PMID)” possibilitou a rastreabilidade dos artigos, enquanto as variáveis “título”, “periódico” e “data de publicação” forneceram informações contextuais essenciais. As variáveis “número de citações de revistas brasileiras” e “número de citações do JVB” foram calculadas para quantificar a representatividade das publicações brasileiras, e a variável “referências” permitiu uma análise detalhada do conjunto de citações utilizadas.

### Verificação e validação dos dados

Para assegurar a qualidade e a integridade dos dados extraídos, o procedimento automatizado foi complementado por verificações manuais realizadas por, pelo menos, dois autores. Esse processo envolveu a conferência cruzada dos dados presentes na planilha com as informações disponibilizadas no PubMed e no NLM Catalog, garantindo que as categorias e os valores extraídos fossem consistentes e corretos.

### Definição e operacionalização das variáveis

Número de citações de revistas brasileiras: refere-se ao total de citações oriundas de periódicos brasileiros presentes nas referências dos artigos analisados. Número de citações do JVB: corresponde à contagem das citações provenientes do próprio JVB.

### Autocitações

A análise de autocitações concentrou-se na identificação dos casos em que o autor principal do JVB citou um artigo publicado em revista brasileira do qual ele próprio era autor, permitindo avaliar a incidência do fenômeno na amostra.

### Apresentação dos dados

Os dados foram apresentados de forma descritiva, utilizando gráficos e tabelas para facilitar a visualização e a interpretação dos achados. Não foram realizadas análises estatísticas inferenciais, visto que o objetivo principal consistia na síntese descritiva dos dados extraídos.

### Limitações e considerações

Uma limitação importante deste estudo é o fato de que o PubMed não indexa todos os artigos publicados pelo JVB desde sua primeira edição. Dessa forma, a amostra pode não refletir a totalidade da produção do periódico, o que pode influenciar a interpretação dos resultados. Além disso, a extração automatizada dos dados, apesar de complementada por verificações manuais, está sujeita a eventuais inconsistências ou erros de indexação.

### Ética

Dispensa de avaliação pelo Comitê de Ética em Pesquisa/Comissão Nacional de Ética em Pesquisa (CEP/CONEP): conforme o Sistema CEP/CONEP, pesquisas que utilizam apenas dados bibliográficos extraídos de bases públicas não necessitam de parecer prévio.

## RESULTADOS

Ao todo, foram extraídos 582 artigos publicados entre os anos de 2016 e 2024, totalizando 13.633 referências. Observando-se a distribuição, não há um padrão consistente de aumento ou diminuição linear do número de publicações por ano: houve um aumento até 2021 (chegando a 110 artigos), seguido de uma redução no triênio 2022-2024, com comportamento predominantemente errático.

Entre o total de referências, 1.300 (9,5%) eram de revistas nacionais, sendo 613 (4,5%) citações do JVB. As citações de revistas brasileiras estavam altamente concentradas em 79 periódicos, entre os 372 indexados ao PubMed.

O número de citações de revistas nacionais por ano manteve-se relativamente estável, com uma média de 2,2 por trabalho publicado no JVB. O número de citações do JVB também apresentou um comportamento estável, com uma média de 1,1 por artigo publicado no JVB (Tabela 1).

**Tabela 1:** Evolução das citações nacionais, autocitações e impacto por artigo no JVB (2016-2024).

Ano	Citações nacionais	Citações JVB	Autocitações JVB	Autocitações (%)	N de artigos	Citações nacionais/artigo	Citações do JVB/artigo
2016	108	54	11	20	45	2,4	1,2
2017	109	49	10	20	59	1,8	0,8
2018	120	50	7	14	61	2,0	0,8
2019	177	87	12	14	67	2,6	1,3
2020	181	88	15	17	75	2,4	1,2
2021	225	103	9	9	110	2,0	0,9
2022	144	74	6	8	58	2,5	1,3
2023	148	66	6	9	66	2,2	1,0
2024	88	42	4	10	41	2,1	1,0

JVB = Jornal Vascular Brasileiro.

Ademais, foram observadas 80 (13%) autocitações referentes ao primeiro autor entre as 613 citações do JVB. Essa taxa diminui progressivamente ao longo dos anos, sendo 20% em 2016 e chegando a 8% em 2022 (Figura 1).

**Figura 1:** Número de citações e autocitações do JVB ao longo dos anos. JVB = Jornal Vascular Brasileiro.

Os periódicos nacionais mais citados foram, em ordem de prevalência: JVB, Revista Brasileira de Ortopedia (RGO), Arquivos Brasileiros de Cardiologia, Revista do Colégio Brasileiro de Cirurgiões, Revista da Associação Médica Brasileira, Revista Brasileira de Cirurgia Plástica, Jornal Brasileiro de Pneumologia, Acta Cirúrgica Brasileira, Radiologia Brasileira e Cadernos de Saúde Pública. Destacam-se as revistas de cirurgia e áreas afins da cirurgia vascular, como cardiologia e pneumologia. Contudo, o JVB ainda representa um percentual significativo das citações de revistas brasileiras, com 47,1% das citações totais (Figura 2).

**Figura 2:** Citações de revistas nacionais pelos artigos publicados no Jornal Vascular Brasileiro entre os anos de 2016 e 2024. J VASC BRAS = Jornal Vascular Brasileiro; RGO = Revista Brasileira de Ortopedia; ARQ BRAS CARDIOL = Arquivos Brasileiros de Cardiologia; REV COL BRAS CIR = Revista do Colégio Brasileiro de Cirurgiões; REV ASSOC MED BRAS = Revista da Associação Médica Brasileira; REV BRAS CIR = Revista Brasileira de Cirurgia Plástica.

A maioria dos artigos analisados utilizou entre 0,01 e 25,00% de citações nacionais em suas referências. No total, 189 (32,00%) publicações analisadas não utilizaram nenhuma referência publicada em revistas nacionais indexadas ao PubMed. Apenas 11 (1,90%) artigos utilizaram mais da metade de referências nacionais, e nenhum utilizou todas as referências oriundas de artigos brasileiros (Tabela 2).

**Tabela 2:** Distribuição percentual de artigos citando revistas nacionais no JVB.

**% de citações de revistas nacionais**		
**0%**	189	32%
**0,01-25%**	323	55%
**25-50%**	59	10%
**51-75%**	10	2%
**> 75%**	1	0%

JVB = Jornal Vascular Brasileiro.

## DISCUSSÃO

O presente estudo parte, como toda ciência, de bases preestabelecidas por colegas interessados nos mesmos tópicos. O termo *voyeurismo* científico foi cunhado por Goffi, no sentido do desprestígio dos periódicos nacionais em favor dos internacionais. Essa prática não apenas desmerece os autores e o esforço de avanço científico brasileiro, como também impede o desenvolvimento das revistas publicadas nacionalmente em relação às suas congêneres, enfraquecendo a imagem e o desenvolvimento da ciência brasileira como um todo^3^.

Um estudo brasileiro realizado pelo Colégio Brasileiro de Cirurgiões, em 2012, indicou um percentual de 11,65% de citações nacionais entre 7.000 referências de três proeminentes revistas de cirurgia geral, o que é compatível com o percentual total de citações encontrado no presente estudo. A maioria das publicações do JVB concentrou-se na faixa de 0,01 a 25,00% de citações nacionais, e apenas 11 artigos citaram mais referências nacionais do que internacionais em sua discussão^4^.

Artigos publicados na área de ortopedia também destacam essa situação há mais de 30 anos, apontando que a RBO apresenta um baixo percentual de citações a referências nacionais em relação a outras revistas da área, mesmo nos artigos publicados na própria RBO^5^. A publicação deste artigo suscitou diversas discussões na área, e cartas ao editor foram enviadas com o intuito de compartilhar a experiência de autores nacionais preteridos em relação a publicações internacionais sobre o mesmo tópico^6^.

Cabe destacar que a taxa de autocitações reduziu progressivamente, caindo de 20 para 8% ao longo dos anos estudados. Isso pode evidenciar o desenvolvimento de um ecossistema científico não predatório, no qual autores publicam e trocam citações, ampliando o alcance das pesquisas nacionais.

A decisão de publicação em uma revista perpassa diversos fatores. Contudo, métricas como o fator de impacto (FI) ocupam um peso desproporcional no meio acadêmico, sendo a principal ferramenta utilizada para a avaliação da qualidade de revistas científicas^7^. Consequentemente, publicações em revistas de alto FI tornam-se símbolos de “*status* científico”, desviando artigos originais de serem publicados nas revistas mais apropriadas^8^.

Estudos internacionais indicam que a preferência por periódicos de alto impacto é um fenômeno global. Fatores como a acessibilidade, o idioma e as estratégias editoriais influenciam significativamente os padrões de citação. Periódicos publicados em inglês e com acesso aberto tendem a alcançar maior visibilidade, enquanto publicações regionais podem sofrer com menor reconhecimento e, consequentemente, menor índice de citações^9^.

Diversas maneiras de se combater esse fenômeno foram discutidas ao longo dos anos. Algumas revistas chinesas, frente ao enorme contingente científico do país e à baixa acessibilidade do idioma, passaram a publicar artigos simultaneamente em dois idiomas: mandarim e inglês. Isso aumenta a circulação local das informações e prestigia as organizações chinesas, ao mesmo tempo em que quebra barreiras para pesquisadores internacionais que desejam se aprofundar nos temas de estudo e citar as publicações^10^. Cabe ressaltar que isso sempre foi feito pelo JVB, que publica todos os artigos em português e inglês.

Campanhas incentivando a publicação de artigos nas revistas nacionais também foram realizadas por sociedades médicas, como a SBACV. Contudo, embora tenham gerado impacto social, não existem dados disponíveis sobre a real efetividade dessa estratégia em relação ao número de citações e publicações dos trabalhos nacionais.

Sabendo da atratividade das revistas internacionais, cabe aos periódicos nacionais estimular estratégias como, por exemplo, a divulgação dos artigos em redes sociais ou mesmo a premiação de autores através de parâmetros como o número de citações.

A baixa citação de autores brasileiros no JVB é multifatorial: reflexos de uma preferência cultural por periódicos estrangeiros (“complexo de vira-lata”), diferenças no nível de evidência dos estudos nacionais em comparação a estudos prospectivos e randomizados estrangeiros, e a necessidade de um fator de impacto competitivo (FI > 2,0) para instaurar um ciclo virtuoso de visibilidade e citações. Esse padrão corrobora estudos prévios na área^11^, que também evidenciaram a sub-representação de autores nacionais em periódicos de cirurgia brasileiros.

## CONCLUSÃO

Este estudo avaliou 13.633 referências extraídas dos artigos do JVB publicados entre 2016 e 2024, e constatou que apenas 1.300 (9,5%) são oriundas de periódicos brasileiros, das quais 613 (4,5%) correspondiam ao próprio JVB. Observou-se, ainda, uma tendência decrescente nas autocitações, o que sugere uma diversificação nas fontes referenciadas. Esses achados evidenciam, de forma descritiva, o perfil das citações utilizadas no JVB e apontam para a necessidade de estratégias que ampliem a visibilidade dos periódicos brasileiros na literatura científica.

## Data Availability

The data supporting the findings of this study are available in the Pubmed repository (https://pubmed.ncbi.nlm.nih.gov/).
